# P-1216. Comparison of the *in vivo* Efficacy and Resistance Development Potential between Cefiderocol (FDC) and Ceftolozane/tazobactam (C/T) Human Simulated Exposures against *Pseudomonas aeruginosa i*n 72 Hour Murine Thigh Infection Model

**DOI:** 10.1093/ofid/ofae631.1398

**Published:** 2025-01-29

**Authors:** Aliaa Fouad, Samantha Nicolau, Pranita Tamma, Patricia J Simner, David P Nicolau, Christian M Gill

**Affiliations:** Hartford Hospital, Farmington, Connecticut; Sanofi, Boston, Massachusetts; Johns Hopkins School of Medicine, Baltimore, MD; Johns Hopkins School of Medicine, Baltimore, MD; Hartford Hospital, Farmington, Connecticut; Hartford Hospital, Farmington, Connecticut

## Abstract

**Background:**

*Pseudomonas aeruginosa* with difficult-to-treat resistance (DTR-PSA) is a clinical burden. C/T is recommended for DTR-PSA although FDC represents an attractive option due to its *in vitro* potency against isolates with C/T-resistance. Head-to-head data assessing these compounds against DTR-PSA are lacking.

**Methods:**

The efficacy and resistance development of FDC and C/T was assessed in a 72h murine thigh model against five clinical DTR-PSA isolates that previously developed resistance to C/T in patients. Index isolates (FDC and C/T-susceptible) were used. Human-simulating regimens (HSR) of FDC 2g IV q8h and C/T 2/1g IV q8h were utilized. Efficacy was assessed as change in bacterial density from starting inoculum and compared with translational endpoints of 1- and 2-log_10_ kill at 24, 48, and 72h. Development of resistance was a post-exposure MIC increase greater than 4-fold doubling dilutions versus untreated controls.

**Results:**

All isolates established a robust infection in the model as the mean ± SD bacterial burden across the 5 test isolates of 5.59 ± 0.4 log_10_ cfu/thigh at 0h and grew over the initial 24h in untreated controls with a mean increase of 3.41 ± 0.88 log_10_ cfu/thigh. FDC reached the 24h 1-log_10_ kill endpoint indicative of clinical efficacy in 5/5 tested isolates, however; C/T reached same endpoint in 3/5 isolates. FDC reached 2-log_10_ kill in all isolates by 48h which was sustained over 72h. Conversely, C/T achieved 2-log_10_ kill 3/5 and 4/5 isolates by 48 and 72h, respectively. In the FDC and C/T-treated groups 17% and 8% of the cultures displayed bacterial eradication after exposure to the HSR regimens which hindered MIC testing for those samples. Development of resistance was not observed post-exposure for either agent.

**Conclusion:**

While frank resistance did not develop to either compound, differences in the rate and extent of kill were observed. Despite susceptibility to both FDC and C/T, humanized FDC exposures provided a more rapid killing and achieved a greater magnitude of bactericidal activity relative to C/T. Since head-to-head randomized comparisons of these agents against DTR-*P. aeruginosa* are unlikely to be conducted, *in vivo* models with humanized exposures provide translational assessment of a challenging clinical scenario.

**Disclosures:**

**Samantha Nicolau, PhD**, Sanofi Pasteur: Full time research scientist **Patricia J. Simner, PhD**, Affinity Biosensors: Grant/Research Support|BD Diagnostics: Grant/Research Support|bioMérieux: Grant/Research Support|Entasis: Personal fees|GeneCapture: Personal Fees|Merck: Personal fees|OpGen Inc: Grant/Research Support|Qiagen: Grant/Research Support|Shionogi: Personal fees|T2 Biosystems: Grant/Research Support **David P. Nicolau, PharmD**, CARB-X: Grant/Research Support|Innoviva: Grant/Research Support|Innoviva: Honoraria|Merck: Advisor/Consultant|Merck: Grant/Research Support|Merck: Honoraria|Pfizer: Advisor/Consultant|Pfizer: Grant/Research Support|Pfizer: Honoraria|Shionogi: Advisor/Consultant|Shionogi: Grant/Research Support|Shionogi: Honoraria|Venatorx: Grant/Research Support **Christian M. Gill, PharmD**, Cepheid: Grant/Research Support|Entasis: Grant/Research Support|Everest Medicines: Grant/Research Support|Shionogi: Grant/Research Support

